# Hijab or Niqab Interacts with Facemasks Usage at Healthcare Settings in Kabul, Afghanistan: A Multi-Center Observational Study

**DOI:** 10.3390/healthcare10101946

**Published:** 2022-10-05

**Authors:** Arash Nemat, Tamim Jan Danishmand, Mohammad Yasir Essar, Nahid Raufi, Shoaib Ahmad, Suleman Lazarus

**Affiliations:** 1 Kabul University of Medical Sciences, Kabul 1001, Afghanistan; 2 Karolinska Institutet, K9 Global Public Health, 17177 Stockholm, Sweden; 3 Ministry of Public Health, Kabul 1001, Afghanistan; 4 District Head Quarters, Teaching Hospital, Faisalabad 37000, Pakistan; 5 Department of Social Policy, London School of Economics and Political Science, Houghton Street, London WC2A 2AE, UK

**Keywords:** facemask usage, gender differences, hospital, COVID-19, Coronavirus, niqab or hijab, preventive behavior, dress code

## Abstract

**Purpose:** We aimed to understand the extent of facemask usage resulting from the third wave of the COVID-19 pandemic in an Afghan context. In Afghanistan, new COVID-19 variants, low vaccination rates, political turmoil, and poverty interact not only with the third wave of the COVID-19 pandemic but also with facemask usage. **Methods:** We collected data (*n* = 1970) by visually observing the usage and type of facemasks used among visitors entering healthcare facilities in Kabul. We conducted an observational study observing the use of facemasks among 1279 men and 691 women. **Results:** While 71% of all participants adhered to wearing facemasks, 94% of these users wore surgical masks, and 86% wore all types of facemasks correctly. Interestingly, women adhered to wearing facemasks more than men. Specifically, of all the participants who were not wearing masks, 20% were men, and only 8% were women. Even though men were more in number in our study (64.9%), women have a higher adherence rate to wearing facemasks than men. **Conclusions:** We conclude that gender socialization and expectations of women to wear the niqab or hijab interact with their adherence to wearing facemasks. Additionally, since Afghanistan is one of the poorest countries in the world, which has witnessed a considerable period of political turmoil, we spotlight that our findings are rare in scholarship as they represent a distinct non-Western Islamic society with a low scale of COVID-19 vaccination. Therefore, more research is needed to assess the general population’s socioeconomic and geopolitical barriers to facemask use, given that Afghanistan is an underrepresented social context. Our findings are expected to aid health policymakers in developing novel prevention strategies for the country.

## 1. Introduction

The Coronavirus Disease 2019 (COVID-19) is an acute respiratory illness that spreads through respiratory droplets and close contacts [1,2,3]. Almost in all world regions, COVID-19 necessitated wearing facemasks to the general population, at least between 2020 and 2022 [1,4]. However, wearing facemasks has not been, and never would be, a uniform experience across nations due to cultural, gender, social, economic, and geopolitical forces [4,5]. Indeed, the COVID-19 pandemic has exposed and exacerbated multiple strands of inequalities worldwide [6,7], including in Afghanistan. Therefore, context matters in discussions of facemask usage. The study aims to examine facemask usage in Afghan society resulting from the advent of COVID-19. While some researchers have focused on COVID-19 issues in Afghanistan [8,9,10], these studies are scanty. Insofar as Afghanistan is one of the poorest nations in the world, findings from this research will be invaluable in the global discussions of facemask usage and other consequences of COVID-19 in general.

The Coronavirus Disease 2019 (COVID-19) pandemic continues to be a public health threat that has raised imminent global concerns. Over 531,690,756 cases of the pandemic have been registered, and 6,310,920 people have died, according to recent statistical data [11]. The SARS-CoV-2 infection is an acute respiratory illness that spreads through respiratory droplets, e.g., through sneezing, coughing, and close contact. Individuals infected by the virus tend to show varying symptoms ranging from fatigue, dyspnea, headache, sneezing, sore throat, fever, cough, and diarrhea [12]. In some cases, the virus might not show any symptoms, making it more dangerous and resulting in community outbreaks and rising infection rates.

During the early stages of the pandemic, countries across the globe enforced lockdowns to contain the spread of the virus. Precautionary measures such as wearing masks were mandatory in preventing infectious outbreaks [13]. Healthcare workers were also advised to follow preventive measures, such as adhering to Personal Protective Equipment (PPE). Enforcing lockdowns and implementing preventative measures have helped many countries contain the rapid spread of the infection. In some countries, wearing a mask was associated with a considerable decrease in cases [14].

Vaccination is an effective public health tool in preventing infectious diseases in the community. Throughout previous outbreaks, vaccines have proven effective in managing the spread of deadly diseases. For instance, during the measles outbreak, the measles vaccination rollout was correlated with a considerable decrease in cases [15]. As in the case of the COVID-19 pandemic, scientists and researchers across the globe have made tremendous efforts to develop different vaccines to contain the pandemic [16]. 

Currently, 10 COVID-19 vaccines have been approved by the World Health Organization (WHO) for use among the population [17]. However, unequal distribution of vaccines has caused significant setbacks for countries with limited resources [18,19]. Many low-income countries have entered the third wave of the pandemic. To effectively manage the pandemic, relying on preventive measures is critical. Among these, facemasks are an effective tool that can significantly help reduce the spread of the virus [20].

In Afghanistan, the COVID-19 pandemic is a significant public health challenge. As the country is battling the third wave of the pandemic, there is an overwhelming shortage of healthcare supplies and workers to mitigate this crisis. Lack of oxygen, shortage of beds, new COVID-19 variants, low vaccination rates, and poverty are some of the critical factors interacting with the third wave [21,22]. Moreover, the recent withdrawal of support from the United States and international allies has wreaked havoc on the socioeconomic burden of the collapsing healthcare system. The healthcare system has stopped functioning at total capacity with little funding and support [23,24]. At this juncture, we carried out an observational study to observe the usage of facemasks among individuals visiting health settings in Kabul. The study aims to highlight the extent of facemask usage among individuals during the third wave of the pandemic. Moreover, the findings of this research could be used to improve preventive measures and prioritize the needs of managing the COVID-19 pandemic in healthcare settings that serve as a hub for treatment and crowded infectious outbreaks simultaneously. 

### 1.1. Objective

This study aimed to investigate the prevalence of facemask usage among individuals visiting hospitals during the COVID-19 pandemic. Additionally, it also sought to determine people’s behavior and perceptions towards preventive measures in hospitals. The findings will provide additional insight to national and international organizations regarding the situation to take timely action and work towards improving hospital management, resource implementation, and public health policies in Afghanistan.

### 1.2. Ethics Approval

The authors obtained ethical approval to conduct the study from the Research Committee of the Microbiology Department of Kabul University of Medical Sciences (DM-RC-21-711). 

## 2. Methods

### 2.1. Study Setting

This prospective observational study was conducted in Kabul, Afghanistan, between June 2021 to July 2021, with the primary objective of assessing the usage of facemasks among individuals visiting the hospital through non-participant observation. A total of 1970 visitors were observed. Patients, their companions, or visitors entering the hospital will be referred to as individuals for this study or participants. The participants were observed inside the hospital departments (indoor) and surrounding yards of the hospital (outdoor) using de-identified patient details without any relation to symptoms presenting for COVID-19, which in turn provided a sample size representative of the general hospital visitors. 

### 2.2. Inclusion and Exclusion

The inclusion criteria for this study were all individuals entering the treatment facility from selected entry points. Exclusion criteria for this study were individuals less than 13 years old, those visiting the emergency department, arriving in an ambulance, working at the treatment facility, individuals suspected of multiple entries, and those exiting the treatment facility entrance.

### 2.3. Data Variables and Data Collection

Data were collected by visually observing the usage and type of facemasks used among visitors entering the 19 treatment facilities. The following demographic data were collected: patient’s gender and age group, location of the participants inside the building of the hospitals and outside the building (indoor and outdoor), time of observation, mask usage, type of masks, and the approach of mask usage. 

During the data collection phase, five observers with experience in healthcare and behavioral sciences were recruited for this study and positioned at different indoor and outdoor locations throughout the hospital campus. The principal investigator conducted a one-hour training session to instruct observers on observation methods, participants' selection, and the study checklist's completion. To ensure the quality of data collection, a senior supervisor accompanied the observers during the first two hours of the observation in the hospital. Supervisors reviewed checklists filled out by observers, and appropriate feedback was provided. 

To prevent the Hawthorne effect, the observation stations were allocated where the subjects could not notice the observers. In contrast, the observers could closely watch the subjects and document relevant findings. For additional accuracy, at the specific time, while the door between the department and yard was closed, both teams started the observation; this prevented double observation of the same subject by both groups. 

Data variables collected include the subjects’ gender, approximate age, usage of facemask, type of facemask, and approach to the use of facemask. Insufficient coverage of the mouth and nose and wearing the facemask upside-down or inside-out were defined as “incorrect or unacceptable” ways of facemask usage. If a mask type was not distinguishable, the observers were not included in the study. 

### 2.4. Statistics

The data was summarized using [Microsoft Excel 2019] and Statistical Package for Social Studies (SPSS) version 25.0 for analysis. Descriptive statistical measures were used to describe the data, including mean, standard deviation, and associated correlations. The estimated prevalence rates were presented with a 95% confidence interval. Chi-square analysis was used to assess the relationship between categorical variables. A *p*-value of <0.05 was considered statistically significant at a 95% confidence interval.

## 3. Result

### Socio-Demographic Characteristics of Study Participants

Data from 2000 subjects who visited the hospital premises were collected. After excluding the non-acceptable subjects, 1970 was included in the study. Of the 1970 participants, the majority (70.9%) were assumed to be less than 40 years of age based on general appearance. In this observational study, more than half of the subjects (64.9%) were men. The majority of the participants (71.3%) adhered to face masks. The most common type of facemask was the surgical mask (94.9%); Cloth and filtered covers were used by 2.7% and 2.4% of the subjects, respectively. Of all the subjects, 86.1% wore masks correctly, while (13.9%) did not follow the standard approach of mask-wearing. Demographic characteristics of the participants, along with the frequency and approach of wearing masks, are shown in Table 1. 

In Table 2, the frequency of facemask usage based on the different hospitals in Kabul is clearly shown. The highest prevalence of facemask usage was seen in two private hospitals: MILAT (95.3%) and KHATUMUL NABEYEEN (91.9%).

**Figure 1:**
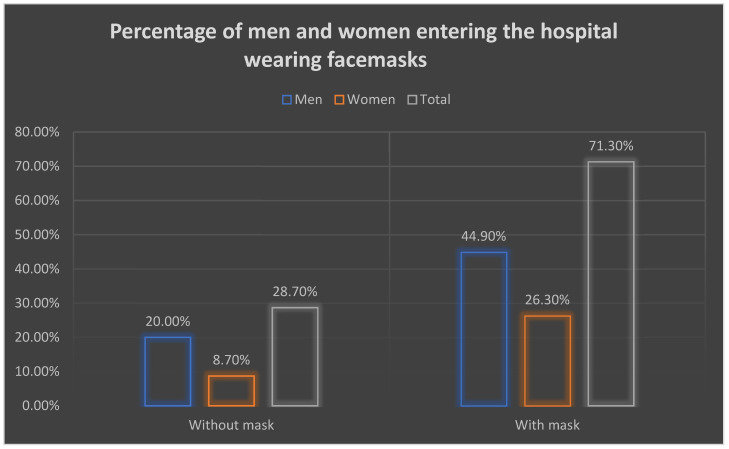
Percentage of men and women entering the hospitals wearing masks.

In terms of gender association with mask usage among participants, from those (71.3%) who wore masks, over 44.9% were men, and only 26.3% were women (illustrated in Figure 1). Among those subjects (28.7%) who were not wearing masks, 20% were men, and only 8.70% were women, as shown in the figure below. 

Regarding gender, women adhered to wearing facemasks more than men. However, there was no significant association between age and mask usage. Inside the ward, more participants were wearing masks compared to outdoor premises. Regarding mask usage, most participants (71.3%) adhered to wearing facemasks, while 28.7% did not. Concerning types of facemasks, the surgical mask was the most prevalent (94.9%) among the participants, while only 2.7% wore a cloth mask. Related to this is that over 86.1% were identified as wearing masks correctly, while 13.9% failed to follow the right approach to mask-wearing. The association between selected socio-demographic variables and mask usage among hospital visitors is shown in Table 3.

## 4. Discussion

This is the first observational study conducted in Afghanistan to evaluate the usage of facemasks among visitors in 19 hospitals in Kabul, Afghanistan. In this study (*n* = 1970), the majority of the participants wore masks. The most common type of mask observed among participants was the surgical mask. Interestingly, women adhered to wearing facemasks more than men. Specifically, of all the participants who were not wearing masks, 20% were men, and only 8% were women. Even though women were less in number in our study, women have a higher adherence rate to wearing facemasks than men. It is noteworthy that during the pandemic, while facemasks are considered an important public health measure for all individuals, different varieties of masks emerged in society. 

While such different types of masks include surgical masks, cotton face masks, N-95 face masks, and cloth face masks, cloth masks are made of various materials with filtering capacities, according to research [23,24]. The CDC has recommended N-95 as the most effective mask for preventing infection. The findings of this study help to highlight the extent of facemask usage among visitors during their visits to the hospitals. Hence, helping policymakers devise plans for preventive measures. 

This study revealed that only two hospitals (Milat and Khatamul Nabeyeen) in Kabul had more excellent management compared to other hospitals in the capital city (Kabul). These two hospitals had the highest number of visitors with masks. This shows that knowledge and experience in hospital management are critical in infection containment. In principle, the general ministry of public health’s policy indicates that during the COVID-19 outbreak wearing a mask is mandatory for the public, particularly in hospitals [10]. However, a good enough health policy is often challenging to implement in general [6,7]. For example, although many Afghans would want to adhere to health policies, most of them face many acute life challenges resulting from abject poverty and a series of political turmoil and conflicts. The recent withdrawal of support from the United States and other international allies also exacerbated these socio-political conditions in Afghan society. 

Consequently, we argue that the socio-economic and political problems mentioned above make it impossible for many Afghans to adhere to the country’s health rules and regulations. To illustrate further, unlike in many world regions, the government cannot provide basic security for the general population due to a series of political turmoil. The advent of the COVID-19 pandemic also compounded such insecurity in Afghan society. Therefore, security issues merged with the low sensitivity to the COVID-19 infection, the high rate of the previous infection, ignorance, and lack of money to buy facemasks were the reasons for breaking the preventive rules for many citizens.

Remarkably, private and government hospitals tend to have distinctive approaches to facemask usage. Some private hospitals aiming to please their affluent visitors do not have strict rules about facemask usage and do not prevent those not wearing masks from entering the hospital premises. On the contrary, governmental hospitals generally provide free healthcare, mainly to poor people who could not afford to use private hospitals. So, it is reasonable to suggest that the poor group of Afghans who visit government hospitals may not be able to afford to be compliant with preventive practices such as mask-wearing due to poverty (see also prior studies) [8,25].

In this study, we found that the proportion of visitors adhering to face masks in hospitals was (71.3%). This is in line with a study conducted in Greece, where 76% of participants declared full or almost full compliance with facemask usage [26]. However, the study in Greece was conducted among the general population through social media platforms. Moreover, a similar study conducted among pedestrians in Iran showed a relatively low (45.6%) facemask usage prevalence [27]. In addition, an observational study conducted among hospital visitors during the COVID-19 pandemic in Malaysia showed that over 96.9% of the participants wore masks [28]. The differences could be described due to the imposed prevention measures in these countries. Afghanistan follows the WHO and CDC recommendations for disease prevention.

Our study observed a significant association between gender and facemask usage. Women were more compliant with facemasks compared to their men. In a study conducted in Malaysia, men/boys between the ages of 18 and 49 were more compliant with facemask usage than women/girls [29]. On the other hand, in a study conducted in Greece, women were more compliant than men with facemask usage [26]. The differences could be due to the cultural, religious, and gender forces in Afghan society, the perceived danger of the virus, and the environment. In Afghanistan, women and girls commonly wear *the niqab* or *hijab* (i.e., face/head covering worn by Muslim women and girls) in public spaces. The wearing of a niqab *or hijab* predates wearing facemasks (e.g., surgical masks) in Afghan society (and other social contexts, for that matter). By implication, women and girls in Afghan communities may perceive the *niqab-wearing* as equal to wearing face masks. Gender and cultural expectations of women and girls are enduring [30,31,32,33,34].

Regarding the *niqab or hijab*, we spotlight that the long-standing cultural, familial, and gender expectations of Afghan women are critical in discussing the wearing of facemasks due to the COVID-19 pandemic. Indeed, Afghanistan is a strongly patriarchal society [30]. So, differential cultural expectations of men and women in society generally socialize men and women distinctively as masculine and feminine individuals, e.g., prescribing distinct acceptable dress codes, according to feminist perspectives [32,33,34,35]. Based on the above insights on gender, cultural and familial forces, we argue that women adhered to wearing facemasks more than men due to gender socialization and expectations of women to be submissive to social forces harmonious to their gender, such as the wearing of *hijabs* or *niqabs* to hide their faces in public spaces. Therefore, these social, gender, familial, and contextual expectations of women (and girls) interact with the adherence to medical prescriptions resulting from the COVID-19 pandemic, in this case, the wearing of the facemask. 

Among the participants, surgical masks were the most prevalent type of masks. This is also observed in other countries [27,36], where most participants indicated wearing surgical masks. This phenomenon could be due to more availability of surgical masks compared to different types of facemasks in society. It could also be due to the affordability of surgical masks over other kinds of facemasks in these regions.

Either way, in this present study, the majority of the participants (86.1%) wore masks correctly. In a similar study conducted in Iran among pedestrians, over 75.6% wore masks correctly [27]. Moreover, in another study conducted through public webcams in the United States, United Kingdom, and Ireland, 51% of the participants wore masks correctly [37]. The critical point is that researchers have shown that the correct and widespread use of facemasks among the general population [38,39] and nurses [40] contributes significantly to limiting the pandemic. 

However, these findings have been observed among pedestrians while our study was conducted solely in healthcare settings, specifically in hospitals in Kabul, during a time of political turmoil. The higher adherence rates in healthcare settings may be due to infection prevention protocols and health policies strictly implemented inside such settings. The higher adherence rates may also be due to social desirability. Afghans, especially women and girls, may interpret adhering to such healthcare policies as presenting themselves in a generally favorable fashion to gatekeepers of the healthcare space as opposed to refusing facemasks usage. Hence, the main strength of our study is that it was conducted in the hospital settings of Kabul, which can provide more significant insights to health policymakers on hospital management and infection prevention. Since Afghanistan is one of the poorest countries in the world, the study’s findings are rare in scholarship as they represent a non-Western Islamic society with a low scale of COVID-19 vaccination. The main strength of our research is also linked to its weaknesses.

## 5. Limitations and Implications

While no study is without weaknesses, this study’s five stands of limitations are as follows. First, the main weakness of our study is that it was conducted in healthcare settings. Therefore, it is unclear whether the participants will practice wearing masks outside such controlled environments. Second, it is noteworthy that we conducted the study during a time of high political turmoil in the country (Afghanistan). Therefore, there may be confounders or other factors associated with the conduct of the study. Thus, conducting similar research in a stable future environment is essential to study the topic more profoundly. Third, this present study was conducted in Kabul, the capital of Afghanistan. Thus, other cities and regions might have a different prevalence of facemask usage. Fourth, the COVID-19 pandemic has exacerbated multiple strands of inequalities worldwide, and Afghanistan is one of the poorest countries in the world. Therefore, using facemasks in wealthy nations in other social or medical environments may not be generalizable. Fifth, given that Afghanistan is a non-Western Islamic nation where face covering (i.e., the wearing of a *niqab or hijab)* is commonplace before the advent of COVID-19, the findings of this study may not travel across cultures. By implication, they could be interpreted as local truths that may not apply to Western societies. 

## 6. Conclusions

This observational study explores using facemasks during hospital visits in Kabul, Afghanistan. The study’s findings are encouraging and call for more research to assess the state of face mask use in the general population. The study highlighted how cultural and gender forces in Afghan society intersect with the adherence to facemask wearing among men and women. Specifically, women have a higher adherence rate to wearing facemasks than men due to gender socialization and expectations of women to wear the *niqab or hijab* (i.e., face/head covering worn by Muslim women and girls in public spaces). Accordingly, the study illuminated that men are less compliant to face mask use than women. Additionally, the study warrants the need for critical assessment and attention to health systems in Afghanistan to aid in strengthening safety precautions during the COVID-19 pandemic. Since Afghanistan is one of the poorest countries in the world, which has witnessed a considerable period of political turmoil, the study’s findings are rare in scholarship as they represent a non-Western Islamic society with a low scale of COVID-19 vaccination. Lastly, our findings are expected to guide health policymakers in developing novel prevention strategies and timely public health interventions in the country.

## Figures and Tables

**Table 1:** Characteristics of participants.

	Frequency	Percent
**Gender**		
Men	1279	64.9
Women	691	35.1
**Age**		
Less than 40	1396	70.9
More than 40	574	29.1
**Location of subject**		
Inside the building of the hospital (indoor)	942	47.8
Inside the building of the hospital (outdoor)	1028	52.2
**Time of observation**		
Morning	867	44.0
Afternoon	1103	56.0
**Mask usage**		
No	566	28.7
Yes	1404	71.3
**Type of mask** (1404)		
Surgical	1333	94.9
Cloth	38	2.7
Filtered (N95)	33	2.4
**Approach of using mask** (1404)		
Correct	1209	86.1
Incorrect	195	13.9

**Table 2:** Mask usage based on the hospital’s visitors.

Hospital Name/Total Observed Subjects	No		Yes	
	Frequency	Percent	Frequency	Percent
**JAMHORIAT/113**	38	33.6%	75	66.4%
**IBNI SINA/115**	34	29.6%	81	70.4%
**MAIWAND/106**	47	44.3%	59	55.7%
**ESTIQLAL/107**	40	37.4%	67	62.6%
**BARCHI/89**	15	16.9%	74	83.1%
**ALI ABAD/110**	32	29.1%	78	70.9%
**ARYANA/104**	19	18.3%	85	81.7%
**AMIRI/105**	18	17.1%	87	82.9%
**ROYAL/90**	17	18.9%	73	81.1%
**GLOBAL/95**	32	33.7%	63	66.3%
**SHAIKH ZAYID/110**	54	49.1%	56	50.9%
**WAZIR AKBAR KHAN/114**	22	19.3%	92	80.7%
**INDRAGANDI/117**	45	38.5%	72	61.5%
**102 BED KHAIR KHANA/111**	34	30.6%	77	69.4%
**SAMA/92**	21	22.8%	71	77.2%
**KHATUMUL NABEYEEN/86**	7	8.1%	79	91.9%
**MILAT/86**	4	4.7%	82	95.3%
**MALALAI/106**	40	37.7%	66	62.3%
**RABEYA BALKHI/114**	47	41.2%	67	58.8%

**Table 3:** Association between selected socio-demographic variables and mask usage among hospital visitors.

Variable *n* = 1970	Mask Usage (By Observation)	
	Yes *n* (%)	No *n* (%)
**Gender**		
Men (1279)	885 (69.2)	394 (30.8)
Women (691)	519 (75.1)	172 (24.9)
	X^2^ = 7.663	*p*₋value = 0.006
**Age**		
Less than 40 (1396)	995 (71.3)	401 (28.7)
More than 40 (574)	409 (71.3)	165 (28.7)
	X^2^ = 0.000	*p*₋value = 0.993
**Location of the subject**		
Indoor/Inside the ward (942)	709 (75.3)	233 (24.7)
Outdoor/In the yard (1028)	695 (67.6)	333 (32.4)
	X^2^ = 14.080	*p*₋value = 0.000
Total	1404 (71.3%)	566 (28.7%)

## Data Availability

The corresponding author can provide data on request.
